# Aroma characteristics of Wuyi rock tea prepared from 16 different tea plant varieties

**DOI:** 10.1016/j.fochx.2023.100586

**Published:** 2023-01-23

**Authors:** Chuan Yue, Hongli Cao, Shaorong Zhang, Zhilong Hao, Zongjie Wu, Liyong Luo, Liang Zeng

**Affiliations:** a College of Food Science/Tea Research Institute, Southwest University, Beibei, Chongqing 400715, China; b College of Horticulture/Key Laboratory of Tea Science in Universities of Fujian Province, Fujian Agriculture and Forestry University, Fuzhou 350002, China; c Wuyi Mountain Yan Sheng Tea Industry Co., Ltd, Wuyishan 354301, China

**Keywords:** Wuyi rock tea (WRT), Aroma profiles, Oolong tea cultivars, Sensory evaluation

## Abstract

Wuyi rock tea (WRT) is famous for its long history and unique characteristic of floral, fruity and nutty flavors. This study investigated the aroma characteristics of WRTs prepared from 16 different oolong tea plant varieties. The sensory evaluation results showed that all WRTs had an ‘Yan flavor’ taste, and the odor was strong and lasting. Roasted, floral and fruity odors were the prime aroma profiles for WRTs. Furthermore, a total of 368 volatile compounds were detected using HS–SPME–GC–MS and analyzed with OPLS–DA and HCA methods. The volatile compounds heterocyclic compounds, esters, hydrocarbons, terpenoids and ketones were the major aromatic components of the WRTs. Specifically, the volatile profiles among newly selected cultivars were comparatively analyzed, and 205 differential volatile compounds were found with variable importance in the projection (VIP) values above 1.0. These results indicated that the aroma profiles of WRTs were mainly dependent on the cultivar specificities of volatile compounds.

## Highlights

• Wuyi rock tea (WRTs) prepared from 16 different oolong tea plant varieties were firstly collected to perform sensory evaluation.
• The volatile compounds of 16 WRT samples were determined using HS–SPME–GC–MS detection and electronic nose.
• The key aroma compounds in the WRT samples were identified, and floral odor volatiles of β-ionone, geraniol, benzene acetaldehyde, benzoic acid, methyl ester and indole were in the top 10 highest rOAVs.
• The aroma characteristics of WRT samples prepared from different cultivars were determined, showing that the flavor of WRT was depended on the cultivar specificities of volatile compounds.

## Introduction

Oolong tea, as one of six types of teas in China, is famous for its exquisite processing, time-honored histories and cultures, healthy function and good taste (Ng et al., 2018, Zeng et al., 2020). Specifically, aroma quality is one of the key characteristics of oolong tea, and oolong teas produced from different cultivars or varieties usually exhibit diverse flavors, which are termed “varieties flavor” (Zhao et al., 2014). High-quality and unique flavors have therefore been persistently pursued by consumers, pushing tea plant breeders to select high-flavor tea plants (Zeng et al., 2020). To date, more than 100 tea plant cultivars or varieties suitable for oolong tea have been selected and used to produce oolong teas, and therefore, the flavor of teas is distinctly produced by different varieties.

Generally, oolong teas are classified into four types, including Wuyi rock tea (WRT), Minnan oolong tea, Taiwan oolong tea and Guangdong oolong tea, according to their sensory quality, production area and technological process (Ng et al., 2018, Zeng et al., 2020). In particular, WRT is a famous oolong tea that is produced through the processing of withering, shaking, rotating, drying and baking from Wuyi city of Fujian Province, China, and has a “Yan flavor”. Dahongpao, Rougui and Shuixian WRTs are the most representative WRTs and enjoy a long reputation at home and abroad. Rougui WRT has a distinctive cinnamon-like aroma, and Shuixian WRT has a flower aroma with woody fragrance (Zhao et al., 2014). Currently, many of the selected oolong tea cultivars or the native tea plant varieties in the Wuyi Mountain area are used to manufacture WRTs, such as Huangmeigui, Huangguanyin, Tieluohan, and Meizhan, which are broadly cultivated in this area, and the WRTs made from these cultivars also have distinctive “variety flavor” characteristics. Recently, the metabolite profile difference among WRT cultivars was analyzed (Chen et al., 2018), and the changes in volatile and nonvolatile compounds during rough processing and final baking were studied extensively using metabolomic and/or transcriptomic analyses (Guo et al., 2021, Liu et al., 2021, Liu et al., 2022, Yang et al., 2021, Yang et al., 2022, Zhang et al., 2019). However, the aroma characteristics of WRTs made from different oolong tea plant cultivars are unclear. In this study, 16 WRTs produced by different oolong tea varieties were used to perform sensory evaluation, and combined with E-nose detection and HS–SPME–GC–MS analysis, the aroma characteristics of WRTs were explored.

## Materials and methods

### WRT sample collection

The major oolong tea plant cultivars, which are suitable for producing WRTs, were planted and managed in the Wuyi Mountain area (Fujian Province, China). The WRTs were produced with traditional processing through sun withering, shaking (five times), fixing, rolling, drying and baking by a tea master with more than 30 years of tea manufacturing experience in YiFa Tea Co., Ltd (Wuyi Mountain City, Fujian, China). Briefly, the fresh tea leaves were withered for 2 ∼ 3 h under sunlight condition (22 ∼ 25 °C and 50 ∼ 55 % humidity). The withered tea leaves were then performed shaking treatment in the WRTs roller machines for five times as following procedure: shaking for 2 ∼ 3 min for the first time, 20 ∼ 30 min for the second time; 40 ∼ 50 min for the third time; 50–60 min for the fourth time; 40 ∼ 50 min for the fifth time; the room air temperature and humidity maintained at 25 ∼ 26 °C and 70 ∼ 75 % respectively. Next, the tea leaves were paned at 180 ∼ 200 °C for 8 ∼ 10 min for fixation. Subsequently, the fixing tea leaves were rolled using the tea twisting machines at room temperature for 8 ∼ 12 min, and then dried in the tea dryer machines for two times under 120 ∼ 130 °C for 15 ∼ 20 min and 110 ∼ 120 °C for 20 ∼ 30 min, respectively. Finally, the dried tea samples were carried out charcoal baking in the bamboo containers for twice, including the 1st baking (temperature 100 ∼ 110 °C, 8 h), and 2nd baking (temperature 120 ∼ 130 °C, 16 h). The WRT samples were subjected to sensory evaluation to identify and ensure their variety-specific characteristics by local tea masters from Yifa Tea Co., Ltd and Wuyi Mountain YanSheng Tea Industry Co., Ltd (Wuyi Mountain City, Fujian, China). The information for the tea samples is listed in Table S1.

### Sensory evaluation

The WRT sensory evaluation was performed according to the standard of China (GB/T 23776, 2018). Briefly, tea infusions from 16 tea plant cultivar samples were prepared by adding 110 mL of boiling water to 5 g of each tea sample in a separate teacup covered with a lid. After brewing for 2 (first cup), 3 (second cup) and 5 (third cup) min, each tea infusion was subjected to a sensory test. The intensity values and aroma descriptors of the samples were verified by ten panelists, which were composed of 6 males and 4 females with an average age of 36, as previously described by Guo et al. (2021). The aroma descriptors of the tea samples were attributed as floral, fruity, fragrance, woody, sweety, nutty, and roasted. The intensities of the aroma attributes were scored using a scale from 0 to 10; the higher the score is, the stronger the intensity, where 0 = none or not perceptible intensity and 10 = extremely high intensity. The first cups were used to evaluate the type of aroma; the second cups were used to determine aroma intensities, and the third cups were used to reevaluate aroma and persistence of aroma. All data are expressed as the average. The second tea infusions from different tea samples were used to evaluate color through a colorimeter as described by Xu et al. (2018). Hunter color values *L^∗^*, *a^∗^*, and *b^∗^* are expressed as the mean value of five scans.

### HS–SPME–GC–MS detection of volatile components

One gram of the powder was transferred immediately to a 20 mL head-space vial (Agilent, Palo Alto, CA, USA) containing NaCl saturated solution to inhibit any enzyme reaction. The vials were sealed using crimp-top caps with TFE-silicone headspace septa (Agilent). At the time of SPME analysis, each vial was placed at 60 °C for 5 min, and then a 120 µm DVB/CWR/PDMS fiber (Agilent) was exposed to the headspace of the sample for 15 min at 100 °C. Desorption of the VOCs from the fiber coating was carried out in the injection port of the GC apparatus (Model 8890; Agilent) at 250 °C for 5 min in splitless mode. The identification and quantification of VOCs was carried out using an Agilent Model 8890 GC and a 5977B mass spectrometer (Agilent) equipped with a 30 m × 0.25 mm × 0.25 μm DB-5MS (5 % phenyl-polymethylsiloxane) capillary column. Helium was used as the carrier gas at a linear velocity of 1.2 mL/min. The injector temperature was kept at 250 °C, and the detector was kept at 280 °C. The oven temperature was programmed from 40 °C (3.5 min), increasing at 10 °C/min to 100 °C, at 7 °C/min to 180 °C, at 25 °C/min to 280 °C, and held for 5 min. Mass spectra were recorded in electron impact (EI) ionization mode at 70 eV. The quadrupole mass detector, ion source and transfer line temperatures were set at 150, 230 and 280 °C, respectively. Mass spectra were scanned in the range of *m*/*z* 50–500 at 1 s intervals. Identification of volatile compounds was achieved by comparing the mass spectra with the data system library (MWGC or NIST) and linear retention index (Metware Biotechnology Co., Ltd., Wuhan, China).

### Volatile compound qualification and rOAV calculation

The contents of volatile compounds were detected according to their peak areas and the peak area of the internal standard compound ([2H]-Phenol). The relative odor activity values (rOAVs) were designed as the ratios of the relative contents and their odor thresholds in water of each volatile compound (Guo et al., 2021, Guo et al., 2022, Xue et al., 2022).

### Electronic nose (E-nose) detection

The aroma profiles of the WRTs were determined using an iNOSE electronic nose (Angshen Intelligent Energy Technology Co., Ltd, Shanghai, China) as previously described by Wu et al. (2022). Briefly, 3.0 g samples were placed into sealed glass vials and stood at 60 °C for 50 min. The volatile compounds were injected into the e-nose at the rate of 0.8 L/min. Sensor resistance was measured during 300 s at the rate of one acquisition every 1 s. For each sample, six replicates were detected. The clusters of volatile compounds were identified by semiconductors S1 (ammonia, amines), S2 (sulfur compounds, terpenes), S3 (hydrogen), S4 (alcohols, organic solvents), S5 (volatile gases produced during food cooking), S6 (methane, biogases, hydrocarbons), S7 (flammable gases), S8 (volatile organic compounds), S9 (oxyhydroxide, gasoline, kerosene), and S10 (alkanes).

### Data analysis of volatile compounds

Unsupervised principal component analysis (PCA) was performed by the statistics function prcomp within R (https://www.r-project.org). The data were unit variance scaled before unsupervised PCA. The hierarchical cluster analysis (HCA) results of samples and metabolites were presented as heatmaps with dendrograms, while Pearson correlation coefficients (PCCs) between samples were calculated by the cor function in R and presented as only heatmaps. Both HCA and PCC were carried out by the R package ComplexHeatmap. For HCA, normalized signal intensities of metabolites (unit variance scaling) are visualized as a color spectrum.

Significantly regulated metabolites between groups were determined by variable importance in the projection (VIP) ≥ 1 and absolute log2FC (fold change) ≥ 1. VIP values were extracted from the OPLS-DA results, which also contained score plots and permutation plots, and were generated using the R package MetaboAnalystR. The data were log transformed (log2) and mean centered before OPLS-DA. To avoid overfitting, a permutation test (200 permutations) was performed.

The statistical analyses were analyzed with one-way analysis of variance (ANOVA) using SPSS (SPSS Inc., Chicago, IL, USA). If the p value < 0.05, the comparisons were regarded as statistically significant.

## Results and discussion

### Sensory profiles of WRTs produced by different varieties

It has been well recognized that the quality of WRTs is characterized by different tea plant varieties. To reveal the variety characteristics of WRT samples, WRTs made by the same tea master using different cultivars’ fresh leaves were collected for investigation (Table S1). The sensory profiles of WRTs made by 16 different varieties were evaluated by an expert panel. The panelists agreed that the tea samples of each type had recognizable typical sensory characteristics. All teas had a bold and twisted, auburn bloom shape (Table S2 and Fig. S1). The tea infusion colors mainly exhibited orange-red and bright color (Table S2 and Fig. S1), and their color values were determined and indicated that the infusion colors were red and yellow (Fig. S1B). All WRTs had strong and lasting aroma and had sweet floral or fruity fragrance (Table S1). The taste of these teas was heavy and mellow, brisk, and had a ‘Yan flavor’, which is the key characteristic of authentic WRT. The leaves exhibited almost red edges. These sensory results indicated that all selected teas had typical characteristics of WRT, which could be used for subsequent research.

### Aroma profiles of WRTs prepared from different tea varieties

**Fig. 1:**
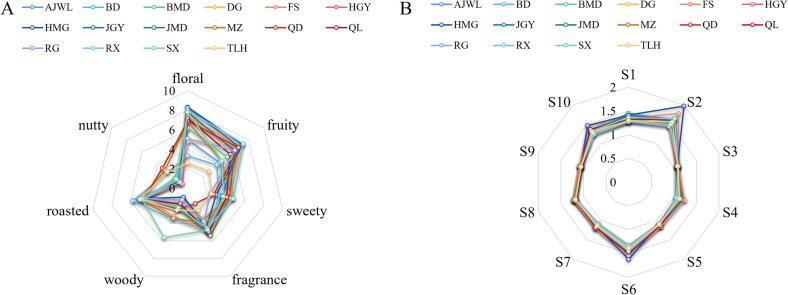
Aroma profiles of 16 WRTs evaluated by sensory analysis (A) and E-nose detection (B).

Aroma profiles determine the selection of suitable oolong tea cultivars (Zeng et al., 2020), and the difference in odors leads to oolong teas being more attractive and mystique. Aroma sensory evaluation was introduced to explore and identify the odor types among 16 WRTs. As shown in Fig. 1A, the odor types of WRTs were described as floral, fruity, fragrance, woody, sweety, nutty, and roasted with different intensities among 16 tea plant varieties. All samples had strong roasted odors and/or subtle nutty aromas, which mainly resulted from baking processing after drying. WRTs of HGY, HMG, JGY, RX, JMD and QD exhibited high floral and fruity odors (scores ≥ 6); all varieties are newly selected tea plant cultivars with high aroma quality and are best for processing into high-quality oolong tea. Specifically, the RG sample had a high fruity odor, whereas SX tea had high floral and woody qualities, both of which are the main planted cultivars used to produce WRTs. Recently, although many oolong tea varieties have been selected and planted to produce WRTs, RG and SX are still the dominant cultivars used to produce WRTs. However, the traditional varieties used to yield WRT, including BD, TLH and AJWL, had low odor intensities of floral, fruity, fragrance and sweet and had high roasted odors.

Additionally, the aroma profiles of 16 WRTs were analyzed using an E-nose and are shown in radar plots. As shown in Fig. 1B, the profiles of aroma among the WRTs exhibited high similarity but with different signal intensities among the ten semiconductors. High signal intensity was detected in S1, S2, S6 and S10, showing that the organic volatiles ammonia, amines, sulfur compounds, terpenes, hydrocarbons and alkanes were significantly enriched, whereas low signal intensity was found in S3 and S9, indicating that hydrogen and oxyhydroxide had low contents in WRTs. Interestingly, several samples of HMG, FS, RX, QL, JMD, DG, AJWL and JGY had high signals in S2, suggesting that these samples may have high terpene contents. The aroma of WRTs prepared with the HMG cultivar has been found to contain a high terpene content (Guo et al., 2022), and terpenes have been recognized as the key aroma compounds contributing to floral and fruity odors in oolong tea (Guo et al., 2021, Liu et al., 2022, Wu et al., 2020, Zhou et al., 2017).

### Volatile compounds identified from WRTs through HS–SPME–GC–MS

**Fig. 2:**
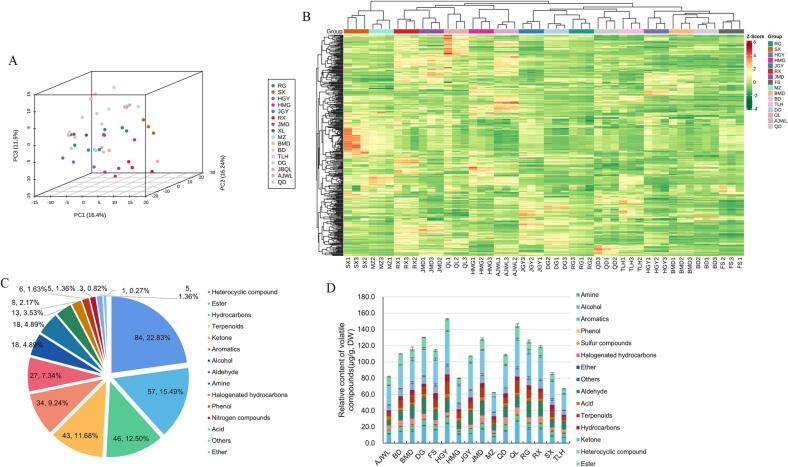
Volatile compound detection and analysis from 16 WRTs. (A) The PCA score plot of different WRTs using all volatile compounds. (B) Cluster heatmap analysis of volatile compounds of 16 WRTs. (C) Categories of determined volatile compounds. (D) The relative amounts of aroma from 16 WRTs quantified to phenol concentration.

The volatile compounds in the WRTs were detected and analyzed using an HS–SPME–GC–MS method. A total of 368 volatile compounds were identified from 16 WRT samples. The PCA score plot and heatmap cluster analysis were performed to identify these compounds. As shown in the PCA plot and heatmap cluster, WRTs were clustered and discriminated from each other according to different tea varieties (Fig. 2A and B), reflecting that the volatile compound profile of each sample was different from the variety used to produce WRTs, and the repeatability of the results of HS–SPME–GC–MS used for volatile compound detection was reliable. The detected volatile compounds were clustered into 15 categories named heterocyclic compounds, esters, hydrocarbons, terpenoids, ketones, aromatics, alcohols, aldehydes, amines, halogenated hydrocarbons, phenols, nitrogen compounds, acids, others and ethers (Fig. 2C). Among these compounds, heterocyclic compounds had the largest number of 84, which accounted for 22.83 % of the total volatile numbers, followed by esters (57), hydrocarbons (46), terpenoids (43) and ketones (34), with proportions of 15.49 %, 12.5 %, 11.68 %, 9.24 % and 7.34 %, respectively, and they accounted for more than 70 % of the volatile compound profile. The remaining 138 volatile compounds belonged to aromatics (27), alcohols (18), aldehydes (18), amines (13), halogenated hydrocarbons (8), phenols (6), nitrogen compounds (5), acids (5), others (3) and ethers (1) with proportions of 4.89 %, 4.89 %, 3.53 %, 2.17 %, 1.63 %, 1.36 %, 1.36 %, 0.82 % and 0.027 %, respectively (Fig. 2C).

As shown in Fig. 2D, the amounts of aroma were investigated by quantifying the phenol concentration. The total contents of all volatiles varied among these WRTs, and the relative contents in RG, HGY, JGY, RX, JMD, FS, BMD, BD, DG, QL and QD were over 100.00 μg/g, of which HGY and QL had higher concentrations, whereas MZ and TLH had lower aroma contents. Furthermore, we investigated the content of volatiles in different classes. Among these volatiles, heterocyclic compounds generally had high contents in all WRTs, followed by aldehydes, alcohols, esters, terpenoids and ketones, which accounted for at least 70 % of the volatile compounds. Correspondingly, the contents of phenols, halogenated hydrocarbons, amines and ethers were lower, with contents under 1.00 μg/g in all WRTs. Notably, we found that HGY, JGY, FS, and MZ had relatively high hydrocarbon contents, whereas relatively low aldehyde contents were observed on FS, MZ, AJWL and QD. Acids, including heptanoic acid, hexanoic acid and butanoic acid, were detected to have high levels in AJWL. Terpenoids are important for aroma quality formation in oolong tea, and we observed that the contents of terpenoids in most WRTs were above 7.00 μg/g, while MZ, TLH and AJWL had relatively low concentrations (Fig. 2D). In contrast, the aroma profiles of other types of teas are different from those of oolong teas. For instance, aldehydes and alcohols are the most abundant volatile compounds accounting for more than 53.7 % of the total volatiles in black teas (Xue et al., 2022), while alcohols, ketones, aldehydes and hydrocarbons are the major volatiles accounting for over 80 % of the total volatiles in brick teas (Zheng et al., 2022), and the major volatile compounds in green tea are alcohols, esters and aldehydes (Cui et al., 2022). Based on the contents and amounts of volatile compounds, in this study, we found that the major types of volatile compounds in WRTs were heterocyclic compounds, aldehydes, alcohols, esters, terpenoids and ketones.

### Identification of key aroma compounds in the WRTs

It has been indicated that compounds with an OAV greater than one are recognized as having key flavor characteristics (Guo et al., 2021, Lee and Noble, 2003). The rOAVs were calculated, and those greater than one were analyzed. In total, 43 rOAVs were above one, and their threshold values and odor descriptions are listed in Table 1. Among these identified compounds, 27 of 43 were classified into terpenoids (10), esters (10), ketones (7), alcohols (4), aldehydes (4), aromatics (3) and heterocyclic compounds (3), and most had floral, fruity, and woody odors. In all WRTs, floral odor volatiles of β-ionone, geraniol, benzene acetaldehyde, benzoic acid, methyl ester and indole had the 10 highest rOAVs, which was consistent with the results of aroma sensory evaluation (Fig. 1). On the other hand, the volatile compounds with roasted or nutty odors of 1-(2-furanylmethyl)-1H-pyrrole and 2,5-dimethyl-pyrazine were also identified with rOAVs above one in all samples; consistently, 2,5-dimethyl-pyrazine has been recognized as a key aroma compound for roasted peanutty flavor in WRTs (Guo, Song, Ho, & Wan, 2018). Previous studies indicated that the baked drying procedure is important for heterocyclic compound generation (Guo et al., 2018, Guo et al., 2021). After this procedure, the numbers and amounts of newly formed heterocyclic compound volatiles were dramatically increased, which contributes to the generation of roasted, nutty, floral and fruity flavors (Chen et al., 2020, Guo et al., 2021). In this study, heterocyclic compounds, such as 2-ethyl-3,5-dimethylpyrazine and 3,5-diethyl-2-methylpyrazine, were reported to be important for roasted odors in oolong tea (Guo et al., 2021, Guo et al., 2022), and we did not detect rOAVs above one in any of the WRT samples. Additionally, linalools, which have been identified as key volatile compounds contributing to oolong tea aroma (Zeng et al., 2020), were also not listed since their rOAVs were less than one because the manufacturing processing and the quality characteristics of WRTs were different from those of Minnan oolong tea (Tieguanyin) or Guangdong oolong tea (Fenghuangdancong). Interestingly, the manufacture processing of sun withering, shaking, and fixing can promote the accumulation of volatile compounds, such as linalool, (E)-nerolidol, indole and jasmine lactone (Hu et al., 2018, Wu et al., 2022, Zeng et al., 2016, Zeng et al., 2018, Zeng et al., 2019, Zhou et al., 2017, Zhou et al., 2021, Zhu et al., 2019, Zhu et al., 2020); drying and baking also contributed to aroma compound formation; however, the contents of certain key floral odor volatiles, including linalool, geraniol, hexanal, and phenethyl isovalerate, were reduced after drying and baking (Guo et al., 2021, Guo et al., 2022, Liu et al., 2022, Yang et al., 2021, Yang et al., 2022), suggesting that baking is the key and fundamental processing for the quality formation of WRTs. Similar to other types of oolong tea, indole, benzoic acid methyl ester, methyl salicylate, methyl jasmonate, (E)-nerolidol, and alpha-farnesene were key contributors to the WRT aroma.

**Table 1:** The volatile compounds with rOAV values greater than one in WRTs produced from 16 varieties.

Compounds	Threshold µg/g#	AJWL	BD	BMD	DG	FS	HGY	HMG	JGY	JMD	MZ	QD	QL	RG	RX	SX	TLH	Odor descriptionψ
β-Ionone	0.000007	3831.15	7259.96	3954.11	8027.46	5158.29	13921.1	3993.47	6172.75	8057.96	3047.55	4223.74	5857.25	5104.68	4694.07	5166.60	4531.52	Floral, sweet, berry-like
Geraniol	0.0007	194.20	711.86	1535.69	605.24	161.26	844.37	860.97	475.57	3381.12	948.19	397.59	3751.60	886.50	1191.00	1323.17	364.33	Rose-like, sweet, honey-like
BenzeneacetAldehyde	0.004	194.70	660.33	767.97	578.44	587.97	727.49	504.91	347.00	598.75	306.93	371.55	566.68	633.94	715.36	361.64	222.66	Floral, rose, cherry-like
2,4-Heptadienal, (E,E)-	0.01	114.56	124.13	206.51	426.19	283.75	213.30	169.81	518.59	552.89	168.52	304.35	388.85	544.87	546.47	227.79	216.08	Fatty, green, oily, cinnamon-like
1-Octen-3-ol	0.001	481.03	551.73	398.61	493.01	515.86	412.47	405.32	407.43	498.91	227.06	454.41	546.47	456.08	422.16	290.35	283.18	Cucumber, earth, fatty, mushroom
4-(2,6,6-Trimethylcyclohexa-1,3-dienyl)but-3-en-2-one	0.00009	563.96	241.55	365.12	477.23	391.48	486.85	234.42	593.11	927.45	224.68	594.70	637.60	452.02	659.30	306.83	395.11	Violet, woody, raspberry
3,5-Octadien-2-one	0.0005	621.74	382.47	366.13	471.73	419.87	488.72	491.63	357.26	744.60	247.93	532.07	502.83	408.33	733.10	210.11	448.30	/
Benzoic acid, methyl ester	0.00052	248.48	336.12	384.16	367.11	365.73	275.65	175.44	316.39	251.68	165.57	454.66	271.13	314.44	320.91	227.66	250.95	Floral
Hexanal	0.0045	44.86	48.31	68.20	83.46	62.96	83.46	73.17	123.12	338.17	53.48	111.28	123.59	101.48	203.65	51.85	92.24	Grassy, green, tallowy
Indole	0.04	60.74	73.91	129.70	36.62	119.21	182.30	51.82	48.80	95.71	29.19	109.45	94.50	59.09	51.55	42.84	32.78	Floral, animal-like
1,3-Cyclohexadiene-1-carboxaldehyde, 2,6,6-trimethyl-	0.003	38.92	53.44	57.59	62.99	47.74	72.38	38.25	50.23	42.88	30.26	36.29	61.05	54.33	44.97	38.50	27.80	Woody, spicy, phenolic
5-Hepten-2-one, 6-methyl-	0.05	36.98	50.80	34.17	60.63	38.40	42.75	40.88	42.05	38.89	21.90	22.35	49.49	49.91	72.26	24.01	17.55	Fruity, apple-like, musty
beta-Myrcene	0.015	11.36	20.14	32.19	30.32	20.85	38.33	20.58	32.75	37.21	16.19	16.43	55.71	42.71	26.76	35.79	13.43	Woody, resinous, musty, balsamic
Methyl salicylate	0.04	29.43	31.02	22.08	43.73	42.91	35.73	25.47	25.56	25.95	12.10	61.61	22.63	39.38	32.93	27.64	23.45	Peppermint, wintergreen mint
d-Limonene	0.034	5.92	5.83	9.19	13.49	10.02	17.91	7.68	15.48	11.97	4.99	8.06	15.25	18.72	8.82	11.99	6.67	Fruity, lemon-like
Phenylethyl Alcohol	0.39	8.56	12.95	19.14	16.46	7.44	10.70	6.61	3.91	17.02	5.33	21.08	14.37	11.83	12.93	5.03	7.63	Floral, rose-like
5,9-Undecadien-2-one, 6,10-dimethyl-, (E)-	0.01	8.03	8.44	10.65	11.16	10.01	17.27	6.93	10.55	15.15	5.22	12.60	13.76	10.89	10.10	9.36	6.80	Flavours and fragrances
2-Cyclopenten-1-one, 3-methyl-2-(2-pentenyl)-, (Z)-	0.007	4.74	4.51	16.97	9.14	9.21	20.61	7.91	8.54	24.46	1.40	23.78	4.74	8.18	5.81	5.34	2.92	Jasmine-like, herbal, floral, woody
Butanoic acid, 2-methyl-, hexyl ester	0.022	7.69	11.64	6.89	14.98	2.42	2.43	2.59	1.28	4.78	4.85	7.76	25.00	7.48	9.67	9.91	3.69	Green, waxy, fruity
BenzAldehyde	0.35	3.56	4.57	4.99	8.89	4.77	5.27	4.16	3.32	4.32	3.28	4.56	5.17	6.87	7.84	3.32	2.92	Caramel, fruity, bitter almond, burnt sugar
alpha-Farnesene	0.087	6.33	20.98	12.03	6.08	13.78	15.78	11.37	4.19	8.45	6.23	3.11	16.39	6.49	8.93	6.20	4.18	Citrus, herbal, lavender-like
Benzeneacetic acid, methyl ester	0.06	3.91	4.07	5.07	6.16	3.62	4.75	2.04	3.07	3.58	2.69	12.38	3.85	5.79	5.26	3.00	6.71	Honey-like
1-(2-furanylmethyl)-1H-Pyrrole	0.1	2.67	5.91	8.06	7.07	3.18	8.34	3.39	10.08	5.11	1.89	6.18	3.15	5.47	4.13	3.35	4.81	Cocoa-like, roasted
Methyl jasmonate	0.003	4.62	8.29	16.27	4.63	7.14	21.53	5.48	3.32	17.80	1.67	6.68	27.62	4.95	7.28	4.10	2.42	Jasmine-like, floral
Naphthalene, 1,2,3,5,6,8a-hexahydro-4,7-dimethyl-1-(1-methylethyl)-, (1S-cis)-	0.0015	12.69	11.19	33.37	6.45	19.82	74.70	25.45	12.89	5.16	5.32	23.70	9.86	4.85	5.15	4.49	8.09	Herbal, woody
beta-Phellandrene	0.036	1.36	1.49	2.64	3.78	2.86	4.36	1.59	5.05	3.17	1.31	2.33	3.83	4.72	2.46	2.74	1.73	Turpentine, terpenic, minty
1,6,10-Dodecatrien-3-ol, 3,7,11-trimethyl-, (E)-	0.25	2.61	4.47	5.38	3.39	4.07	7.61	2.12	1.61	6.03	1.12	3.41	5.35	4.14	4.00	1.63	1.42	Floral, green, citrus, waxy
*n*-Valeric acid *cis*-3-hexenyl ester	0.06	3.90	6.37	4.22	8.64	1.56	3.66	3.85	2.36	9.43	3.05	4.16	5.24	3.86	3.67	3.35	1.72	Fruity, apple, pear, kiwi, banana-like
3-Hexen-1-ol, (E)-	0.11	2.40	6.74	3.68	4.24	4.39	5.33	5.53	2.16	5.19	2.18	2.32	2.70	3.38	4.06	2.46	1.05	Green, moss, cut grass, fresh
2,6,6-Trimethyl-2-cyclohexene-1,4-dione	0.025	1.68	1.47	1.53	2.28	1.46	1.78	1.13	1.50	1.89	0.87	1.42	2.18	2.38	1.89	1.38	1.13	Woody, musty, tea-like
Tridecane	0.042	0.91	1.80	1.95	1.60	2.81	3.47	1.20	2.17	2.06	1.35	2.14	1.83	1.87	1.27	2.34	1.03	Alkane-like
Naphthalene	0.05	1.50	1.56	1.89	1.99	2.07	2.84	1.15	3.30	2.52	0.93	2.50	1.92	1.76	1.35	1.42	1.10	Tar-like
1-Decanol	0.023	0.51	1.02	2.03	1.00	0.61	1.28	0.35	0.30	0.42	0.43	0.98	0.60	1.55	0.45	0.86	0.35	Rose-like
Toluene	0.527	0.93	1.55	1.32	2.10	1.05	1.76	0.75	1.40	1.05	0.72	1.62	1.21	1.47	1.47	1.12	1.33	Chemical, synthetic, solvent, aromatic
Acetic acid, 2-phenylethyl ester	0.24959	0.87	1.13	2.05	1.74	0.57	0.75	0.35	0.32	1.33	0.29	2.33	0.98	1.36	1.45	0.51	0.76	Floral, rosy, with a slight balsamic nuance
Pyrazine, 2,5-dimethyl-	1.75	1.61	1.35	1.26	1.02	1.40	1.16	1.21	1.21	1.03	1.45	1.37	1.48	1.10	1.07	1.38	1.45	Nutty, coffee, cocoa-like
2-Heptanone	0.65	1.13	1.39	1.14	1.15	1.22	1.35	1.02	1.21	0.81	0.46	1.14	0.94	1.02	0.76	0.95	0.65	Truffle, earth, nut
Hexanoic Acid	3	1.21	0.96	0.88	1.03	0.90	1.16	1.04	0.81	1.10	0.35	0.96	0.89	0.95	0.89	0.76	0.44	Sweaty
alpha-Ionone	0.076	0.96	0.93	1.01	0.94	0.93	1.53	0.71	1.12	1.97	0.43	0.75	0.85	0.87	1.04	0.81	0.56	Floral, violet, powdery, berry-like
Butanoic acid, hexyl ester	0.203	1.80	0.89	0.56	0.95	0.50	0.53	0.49	0.20	0.79	0.40	0.41	3.34	0.85	1.03	0.72	0.14	Fruity, green
p-Xylene	1	0.65	0.94	0.77	1.28	0.39	1.03	0.34	0.90	0.56	0.28	1.10	0.70	0.79	0.75	0.63	0.91	Plastic, green, pungent
eta-Phenylethyl butyrate	0.376	1.49	0.63	0.48	0.97	0.18	0.23	0.20	0.14	0.58	0.21	0.61	2.24	0.40	0.55	0.20	0.28	Sweet, floral
Benzoic acid, hexyl ester	0.073	1.01	0.33	0.45	0.43	0.76	0.14	0.17	0.19	0.48	0.83	0.76	1.76	0.31	0.82	0.19	0.41	Fresh, balsam, sappy

# All the odor thresholds were obtained from Guo et al. (2021).

ψ Odor description found in the literature with database (Flavornet; The LRI and Odour Database).

### Volatile compound comparative analysis between varieties

**Fig. 3:**
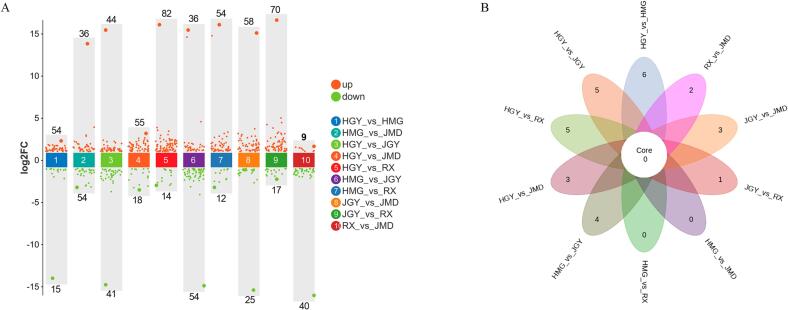
Volatile compound comparative analysis among HGY, JGY, JMD, HMG, and RX. (A) Differential volatile compound (DVCs) analysis among pairwise comparisons, and the numbers of up-and down-regulated DVCs were indicated. (B) Venn diagram analysis of differential volatile compounds among HGY, JGY, JMD, HMG, and RX.

**Fig. 4:**
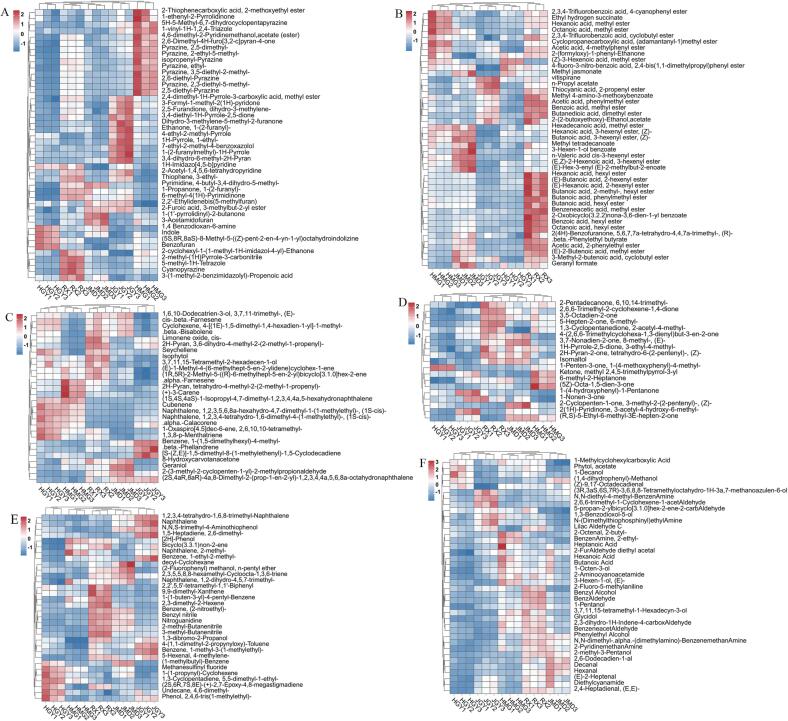
Cluster heatmap analysis of volatile compounds with variable importance in the projection (VIP) values above one in the comparisons of HGY, JGY, JMD, HMG, and RX. (A) heterocyclic compounds; (B) esters; (C) terpenoids; (D) ketones; (E) aromatics, hydrocarbons, halogenated hydrocarbons, nitrogen compounds and others; (F) alcohol, acid, aldehyde and amin.

Since the aroma odors of WRTs were closely related to the difference in varieties, the key cultivars of interest were selected to explore the volatile compounds. In particular, HGY, JGY, JMD, HMG, and RX, which were selected from the filial generation of Tieguanyin and Huangdan or progenies of the natural hybrid of Huangdan, suggesting that they had a close genetic background (Wang et al., 2021), were subjected to pairwise comparison analysis. As shown in Fig. 3A, the differential volatile compounds (DVCs) among the comparisons were significantly varied. HGY_vs_RX exhibited the most DVCs, 96 DVCs, including 14 downregulated and 84 upregulated DVCs, whereas there were 49 DVCs, including 40 downregulated and 9 upregulated DVCs, identified from RX_vs_JMD. In total, 205 DVCs were identified based on VIP > 1, p < 0 0.05, and |log2FC| > 1, and most of them were clustered into heterocyclic compounds (44), esters (43), terpenoids (28), ketones (19), alcohols (14), aromatics (14), and aldehydes (13) (Fig. 4). Interestingly, we found that the proportion of upregulated DVCs in the comparisons of HGY_vs_RX, JGY_vs_RX, HMG_vs_RX and that of downregulated DVCs in RX_vs_JMD were greater than those in the other comparisons, indicating that the volatile compounds of RX were more abundant than those of HGY, HMG, JGY and JMD. Consistently, although the WRTs manufactured from each cultivar have their own characteristics, RX WRTs usually have high floral and fruity fragrant sensory value in practice. In this study, we found that RX had high levels of floral and fruity odor compounds, i.e., benzyl alcohol, benzaldehyde, and hexanoic acid hexyl ester. Specifically, we found that JGY WRTs had high levels of propyl acetate and beta-phellandrene, HGY WRTs had high indole contents, HMG WRTs had high levels of alpha-farnesene, and JMD WRTs had high levels of geraniol and hexanal. Additionally, we generated a Venn diagram to investigate the DVCs shared by those cultivars (Fig. 3B). There were 6 DVCs for HGY_vs_HMG, 5 DVCs for HGY_vs_JGY and HGY_vs_RX, 4 DVCs for HMG_vs_JGY, and 3 DVCs for HGY_vs_JMD and JGY_vs_JMD, and JGY_vs_RX had one DVC, reflecting that the volatile compounds of HGY were greatly different from those of other cultivars. Although we did not detect their parents’ volatiles compounds (Tieguanyin and Huangdan) since they were not been used to produce WRTs, previous studies showed that the oolong tea plant hybrids had volatile heterosis (Wang et al., 2022, Zheng et al., 2019); therefore, higher content and/or special aroma-containing oolong tea plants could be generated through cross breeding using elite precursors.

## Conclusions

In this study, WRTs produced using 16 representative oolong tea varieties were collected and analyzed. The WRTs had a ‘Yan flavor’ taste, and the odor was strong and lasting. The aroma type of WRTs had strong roasted and high floral and fruity odors. A total of 368 volatile compounds were identified and determined using HS–SPME–GC–MS analysis. The volatile compounds heterocyclic compounds, esters, hydrocarbons, terpenoids and ketones were the major aromatic components of the WRTs. The cultivars HGY and QL had higher volatile compound levels than the other cultivars, and the aroma contents of MZ and TLH were lower. The key volatile compounds with rOAVs above one were identified, and most of them had floral, fruity, and woody odors, while floral odor volatiles β-ionone, geraniol, benzene acetaldehyde, benzoic acid, methyl ester and indole had the top 10 highest rOAVs. Additionally, the volatile compounds among WRTs prepared with HGY, JGY, JMD, HMG, and RX were compared, and the key DVCs among comparisons were also identified. The aroma characteristics of WRTs produced from different cultivars were determined. Our results provide a comprehensive landscape for volatile compounds of WRTs produced from 16 representative oolong tea cultivars and are helpful for further scientific understanding WRTs.

## Ethics approval

The study did not involve any human or animal testing.

## CRediT authorship contribution statement

**Chuan Yue:** Investigation, Methodology, Data curation, Formal analysis, Writing – original draft, Writing – review & editing, Funding acquisition. **Hongli Cao:** Data curation, Investigation, Writing – review & editing. **Shaorong Zhang:** Investigation, Writing – review & editing, Methodology. **Zhilong Hao:** Investigation, Methodology, Writing – review & editing. **Zongjie Wu:** Investigation, Methodology. **Liyong Luo:** Data curation, Writing – review & editing. **Liang Zeng:** Conceptualization, Funding acquisition, Supervision.

## Declaration of Competing Interest

The authors declare that they have no known competing financial interests or personal relationships that could have appeared to influence the work reported in this paper.

## Data Availability

The data that has been used is confidential.
