# Supplementary material for: Aroma characteristics of Wuyi rock tea prepared from 16 different tea plant varieties
Source: Food Chem X. 2023 Jan 23;17:100586. doi: 10.1016/j.fochx.2023.100586 (PMC9945420; doi:10.1016/j.fochx.2023.100586)
Supplement: Supplementary data 1 [file mmc1.docx]

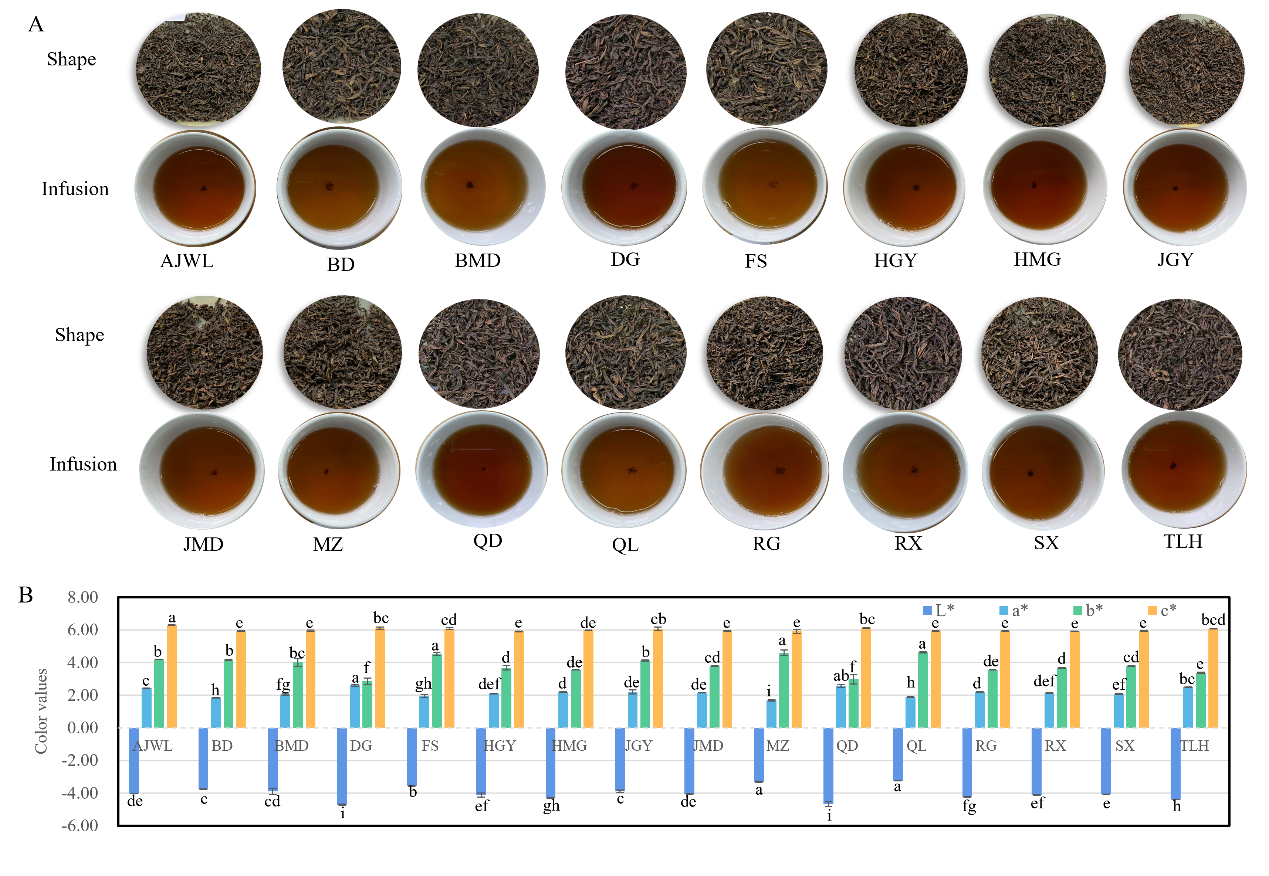


**Fig. S1** The shape, infusion and infusion color values of 16 WRTs used in this study. The shape and infusion of the second brewed cup were recorded (A). The second tea infusions were used to evaluate color using a colorimeter (B). Hunter color values *L^∗^*, *a^∗^*, and *b^∗^* are expressed as the mean value of five scans.

**Table S1** Information of 16 Wuyi rock teas in the Wuyi National Park

| **Samples** | **Full names** | **Location** | **Genetic background** |
| --- | --- | --- | --- |
| AJWL | Aijiaowulong | Dongyuanji (27°52′23″N, 117°56′25″E) | selected from northern Fujian area |
| BD | Beidou | Xianrenyan (27°45′12″N, 118°0′11″E) | selected from northern Fujian area |
| BMD | Baimudan | Yuhuadong (27°42′15″N, 117°59′10″E) | selected from northern Fujian area |
| DG | Dangui | Xianrenyan (27°45′12″N, 118°0′11″E) | selected from RG descendant |
| FS | Foshou | Yuhuadong (27°42′15″N, 117°59′10″E) | selected from southern Fujian area |
| HGY | Huangguanyin | Xianrenyan (27°45′12″N, 118°0′11″E) | generation of Tieguanyin and Huangdan |
| HMG | Huangmeigui | Xianrenyan (27°45′12″N, 118°0′11″E) | generation of HGY and Huangdan |
| JGY | Jinguanyin | Xianrenyan (27°45′12″N, 118°0′11″E) | generation of Tieguanyin and Huangdan |
| JMD | Jinmudan | Lixili (27°45′15″N, 117°57′40″E) | generation of Tieguanyin and Huangdan |
| MZ | Meizhan | Shizifeng (27°38′10″N, 117°58′19″E) | selected from southern Fujian area |
| QD | Qidan | Dongyuanji (27°52′23″N, 117°56′25″E) | selected from northern Fujian area |
| QL | Qilan | Lixili (27°45′15″N, 117°57′40″E) | selected from southern Fujian area |
| RG | Rougui | Bishiyan (27°41′41″N, 117°57′36″E) | selected from northern Fujian area |
| RX | Ruixiang | Lixili (27°45′15″N, 117°57′40″E) | progenies of natural hybrid of Huangdan |
| SX | Shuixian | Lixili (27°45′15″N, 117°57′40″E) | selected from northern Fujian area |
| TLH | Tieluohan | Xianrenyan (27°45′12″N, 118°0′11″E) | selected from northern Fujian area |

**Table S2** Results of sensory evaluation of 16 WRTs

| **Teas** | **Shape** | **Infusion color** | | **Aroma** | **Taste** | **Leaves** |
| --- | --- | --- | --- | --- | --- | --- |
| AJWL | bold and twist, auburn bloom | orange red, bright | strong and lasting, floral, slight pungent aroma | | heavy and mellow, almost fresh and yan flavour | fat, bright, almost red edge |
| BD | straight, auburn bloom | orange red, bright | strong and lasting, floral-fresh | | heavy and mellow, sweet and brisk, alomst yan flavour | fat, soft, bright, almost red edge |
| BMD | bold, auburn bloom | light orange red, bright | strong and lasting, floral-fresh | | heavy and mellow, sweet and brisk, alost yan flavour | fat, soft, bright, almost red edge |
| DG | bold and twist, auburn bloom | deep orange red, bright | strong and lasting, sweet floral and fruity | | heavy and mellow, sweet and brisk, almost yan flavor | fat, soft, bright, almost red edge |
| FS | bold and straight, auburn bloom | orange red, bright | strong and lasting, fruity | | heavy and mellow, sweet and brisk, yan flavour | bold, soft, bright, almost red edge |
| HGY | bold and twist, auburn bloom | orange red, bright | strong and lasting, sweet floral | | heavy and mellow, sweet and brisk, yan flavor | bold, bright, almost red edge |
| HMG | bold and twist, auburn bloom | deep orange red, bright | strong and lasting, sweet fruity | | heavy and mellow, almost yan flavor | bold, soft, bright, almost red edge |
| JGY | bold and twist, auburn bloom | orange red, bright | strong and lasting, sweet floral | | heavy and mellow, sweet and brisk, almost yan flavor | fat, bright, almost red edge |
| JMD | bold and straight, auburn bloom | orange red, bright | strong and lasting, sweet fruity | | heavy and mellow, sweet and brisk, almost yan flavour | fat, soft, bright, almost red edge |
| MZ | bold and straight, auburn bloom | orange red, bright | strong and lasting, floral, pungent aroma | | mellow, sweet and brisk, alomost yan flavour | fat, soft, bright, almost red edge |
| QD | bold and twist, auburn bloom | deep orange red, bright | strong and lasting, sweet fruity | | heavy and mellow, sweet and brisk, yan flavor | fat, soft, bright, almost red edge |
| QL | bold, auburn bloom | light orange red, bright | strong and lasting, floral | | heavy and mellow, sweet and brisk, yan flavour | fat, soft, bright, almost red edge |
| RG | bold and twist, auburn bloom | orange red, bright | strong and lasting, pungent aroma, fruity | | heavy and mellow, sweet and brisk, almost yan flavour | fat, soft, bright, almost red edge |
| RX | bold and twist, auburn bloom | orange red, bright | strong and lasting, sweet fruity | | mellow, sweet and brisk, almost yan flavour | fat, soft, bright, almost red edge |
| SX | bold, auburn bloom | orange red, bright | strong and lasting, sweet floral, woody fragrance | | heavy and mellow, fresh and brisk, alomost cong flavour | bold, soft, bright, almost red edge |
| TLH | bold and twist, auburn bloom | orange red, bright | strong and lasting, fruity | | heavy and mellow, brisk, almost yan flavor | fat, soft, bright, almost red edge |
